# Lichen Planus Induced by Pegylated Interferon Alfa-2a Therapy in a Patient Monitored for Delta Hepatitis

**DOI:** 10.1155/2015/389131

**Published:** 2015-03-03

**Authors:** Safak Kaya, Eyup Arslan, Birol Baysal, Sule Nergiz Baykara, Ozlem Ceren Uzun, Sehmuz Kaya

**Affiliations:** ^1^Department of Infectious Diseases, Gazi Yasargil Training and Research Hospital, Turkey; ^2^Department of Infectious Diseases, Selahaddin Eyyubi State Hospital, Turkey; ^3^Department of Gastroenterology, Bezmialem University Faculty of Medicine, Turkey; ^4^Department of Dermatology, Selahaddin Eyyubi State Hospital, Turkey; ^5^Department of Pathology, Selahaddin Eyyubi State Hospital, Turkey; ^6^Department of Orthopedics Surgery, Van Training and Research Hospital, Turkey

## Abstract

Interferons are used for treatment of chronic hepatitis B. They can induce or exacerbate some skin disorders, such as lichen planus. In this study, as we know, we presented the first case developing lichen planus while receiving interferon treatment due to delta hepatitis. A 31-year-old male patient presented to our outpatient clinic with HBsAg positivity. With his analyses, HBV DNA was negative, anti-delta total was positive, ALT was 72 U/L (upper limit 41 U/L), and platelet was 119 000/mm^3^. He was therefore started on subcutaneous pegylated interferon alfa-2a therapy at 180 mcg/week for delta hepatitis. At month 4 of therapy, the patient developed diffuse eroded lace-like lesions in oral mucosa, white plaques on lips, and itchy papular lesions in the hands and feet. Lichen planus was considered by the dermatology clinic and topical treatment (mometasone furoate) was given. The lesions persisted at month 5 of therapy and biopsy samples were obtained from oral mucosal lesions and interferon dose was reduced to 135 mcg/week. Biopsy demonstrated nonkeratinized stratified squamous epithelium; epithelial acanthosis, spongiosis, and apoptotic bodies were observed in the epidermis and therefore lichen planus was considered. At month 6 of therapy, lesions did not improve and even progressed and interferon treatment was therefore discontinued.

## 1. Introduction

Lichen planus (LP) is an inflammatory disease in which chronic keratosis occurs in the skin or mucous membranes or both. Skin lesions are located in the extremities, genitalia, nails, face, and scalp. Mucous membrane lesions are located in the oral cavity, nasal mucous membranes, throat, esophagus, stomach, bladder, vagina, and glans penis [1]. The incidence in the general population is reported to be 1% [2]. Interferons are used for treatment of chronic hepatitis B, C, and D. They can induce or exacerbate some skin disorders, such as lichen planus, with cytokine cascades [3]. However, there is an association between chronic hepatitis with LP. The first report indicating the association between chronic liver diseases and LP was informed in 1978 [4]. In the literature, there are many reports related to lichen planus development in patients receiving interferon therapy due to chronic hepatitis C. In this study, as we know, we presented the first case developing lichen planus while receiving interferon treatment due to delta hepatitis.

## 2. Case

**Figure 1:**
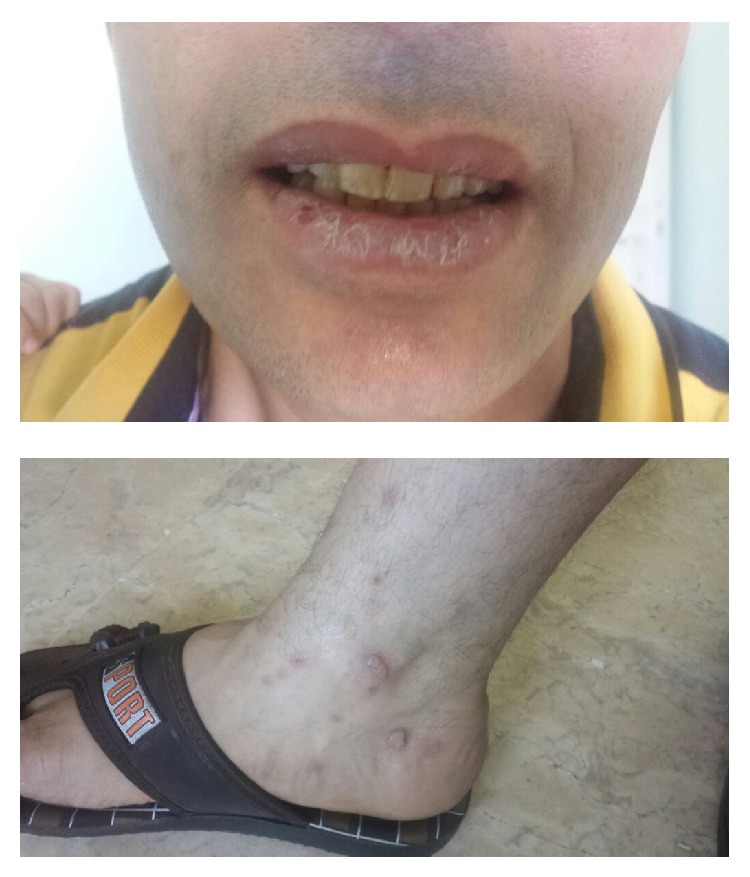
The white plaques on lip and itchy papular lesion in the foot.

**Figure 2:**
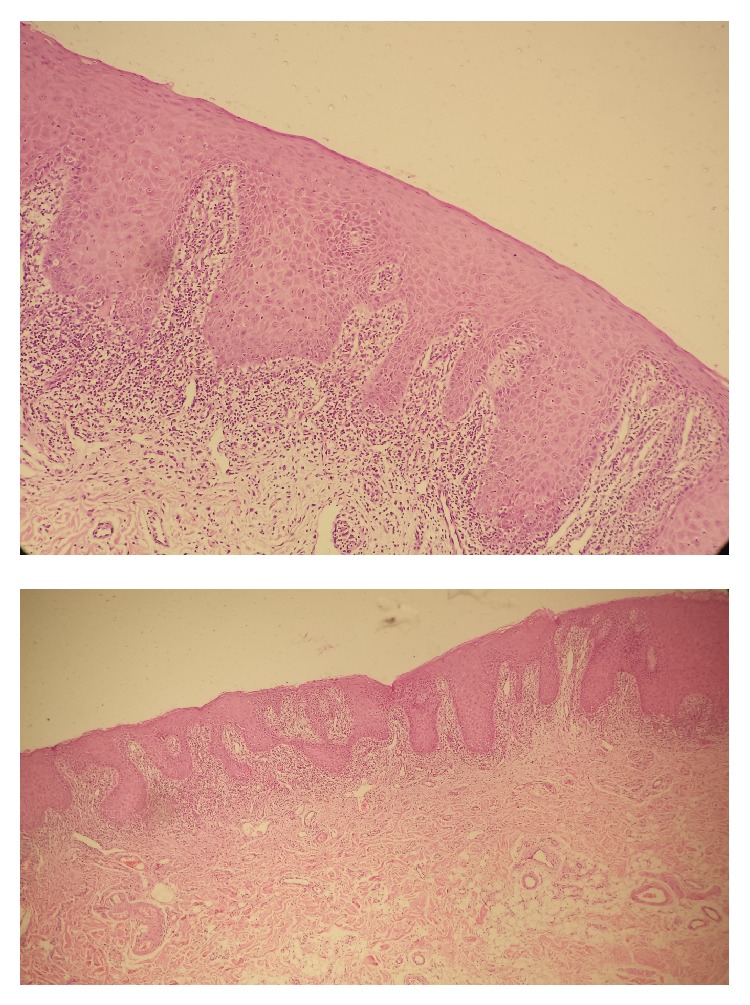
Chronic inflammatory cell infiltration in dermoepidermal junction in the buccal mucosa.

A 31-year-old male patient presented to our infectious diseases outpatient clinic with HBsAg positivity. His only complaint was weakness. He had no underlying diseases. His physical examination yielded normal results. With his analyses, HBV DNA was negative, anti-delta total was positive, ALT was 72 U/L (upper limit 41 U/L), platelet was 119 000/mm^3^. Other whole blood count and biochemistry results were within normal ranges. Liver biopsy was considered, but the patient refused. He was therefore started on subcutaneous pegylated interferon alfa-2a therapy at 180 mcg/week for delta hepatitis. At month 4 of therapy, the patient developed diffuse eroded lace-like lesions in oral mucosa, white plaques on lips, and itchy papular lesions in the hands and feet (Figure 1). Lichen planus was considered by the dermatology clinic and topical treatment (mometasone furoate) was given. The lesions persisted at month 5 of therapy and biopsy samples were obtained from oral buccal mucosal lesions and interferon dose was reduced to 135 mcg. Biopsy demonstrated nonkeratinized stratified squamous epithelium; epithelial acanthosis, spongiosis, and apoptotic bodies were observed in the epidermis. In addition, lymphohistiocytic inflammatory cells were noticed especially in the style of the band dermoepidermal junction. Therefore lichen planus was considered (Figure 2). At month 6 of therapy, lesions did not improve and even progressed and interferon treatment was therefore discontinued. Over the 3-month period following treatment cessation, lesions reduced but persisted.

## 3. Discussion

LP is a major extrahepatic manifestation observed in HCV infection. Studies investigating the pathogenetic basis of the relationship between hepatitis C and LP reported that lichen planus was a disease that is associated with type 1 interferon as HCV and herpes virus infections and that LP may occur or existing lesions may worsen during therapy in diseases treated with interferon such as HCV infection and melanoma [5, 6]. There are several studies on concomitant LP and HCV. There is also a report describing a relationship between interferon and LP in the absence of HCV infection. Interferon alpha can induce LP, the most likely mechanism being the induction of cytokine cascade [7]. Dalekos et al. studied a total of 120 patients treated with interferon alpha for chronic viral hepatitis (of these, 67 were treated with interferon for hepatitis B, 45 for hepatitis C, 6 for both hepatitis B and hepatitis C, and 2 for delta hepatitis) and reported new lichen planus in 2 patients with chronic hepatitis C and in 1 patient with hepatitis B. Neither of the patients with delta hepatitis developed a dermatologic complication [8]. This may be due to the low number of subjects studied. Our patient is the first case of delta hepatitis in the literature developing lichen planus during interferon alpha treatment and is one of the 38 delta hepatitis patients being monitored by us. Although the first connotations of viral hepatitis and LP are HCV and extrahepatic manifestation, it should be borne in mind that LP may occur also with interferon alpha treatment in patients with delta hepatitis.
